# A Novel Approach to Identify Enhancer lincRNAs by Integrating Genome, Epigenome, and Regulatome

**DOI:** 10.3389/fbioe.2019.00427

**Published:** 2019-12-17

**Authors:** Hui Liu, Tiantongfei Jiang, Shuyuan Wang, Xiang Chen, Xiaoyan Jin, Qi Wang, Xinhui Li, Jiaqi Yin, Tingting Shao, Yongsheng Li, Juan Xu, Qiong Wu

**Affiliations:** ^1^College of Bioinformatics Science and Technology, Harbin Medical University, Harbin, China; ^2^Key Laboratory of Tropical Translational Medicine of Ministry of Education, College of Biomedical Informatics and Engineering, Hainan Medical University, Haikou, China; ^3^School of Life Science and Technology, Harbin Institute of Technology, Harbin, China

**Keywords:** enhancer lincRNA, mouse embryonic stem cell, regularized regression model, epigenome, regulatome

## Abstract

LincRNAs enriched with high H3K4me1 and low H3K4me3 signals often have the enhancer-like features which are named as enhancer-associated lincRNAs (elincRNAs). ElincRNAs are considered to be indispensable for target gene transcription, which play important roles in development, signaling events, and even diseases. In this study, we developed a regularized regression model to identify elincRNAs by integrating the genomic, epigenomic, and regulatory data. Application of the proposed method to mouse ESCs reveals that besides the basic well-known epigenetic features H3K4me1 and H3K4me3, more specific epigenetic features, such as high DNA methylation, high H3K122ac, and H3K36me3 were contributed to mark elincRNAs with the best accuracy and precision. Finally, 3729 elincRNAs were identified in mouse ESCs. Furthermore, the elincRNAs and canonical lincRNAs exhibit distinct genomic features, and elincRNAs have the higher CGI enrichment and lower sequence conservation. Through the analysis of transcription regulation, we found that elincRNAs were significantly regulated by NANOG, POU5F1, SOX2 and ESRRB, and were involved in the core transcriptional regulatory circuitry controlling ES cell state Function enrichment analysis further discovered that elincRNAs tended to regulate specific embryonic development biological processes. These results indicated that these two types of lincRNAs had both specific epigenetic and transcriptional regulation mechanism and display distinct functional characters. In conclusion, we presented a credible computational model to prioritize novel elincRNAs, and depicted the atlas of elincRNAs in mouse ESCs, which would help dissect the function roles of lncRNAs during the mammalian development and diseases.

## Introduction

Long non-coding RNAs (lncRNAs) are a class of RNA transcripts longer than 200 nucleotides which are not transcribed into proteins (Derrien et al., 2012). Most lncRNAs are PolII-dependent transcribed, whose transcripts have the exon-intron structure with the 5′ capping and poly-A trail (Dinger et al., 2008). In comparison with mRNAs, lncRNAs generally have lower expression abundance and sequence conservation. Recently, crucial roles of lncRNAs in cell differentiation, embryonic development and complex diseases have been confirmed in multiple studies (Lee and Bartolomei, 2013; Flynn and Chang, 2014; Hamazaki et al., 2015; Zhou et al., 2017, 2018, 2019). Guttmanet al. knocked down 147 lncRNAs expressed in mouse embryonic stem cells (ESCs), which resulted in the significant changes of the global gene expression in mouse ESCs, indicating that lncRNAs had key roles in the circuitry controlling ES cell state (Guttman et al., 2011). LncRNAs can regulate gene expression through a number of mechanisms, across epigenetic, transcriptional, and alternative splicing regulation (Gong and Maquat, 2011; Sun et al., 2014; Tay et al., 2014; Rutenberg-Schoenberg et al., 2016; Zhou et al., 2018). LncRNAs could regulate gene expressions in both cis and trans effects, influencing or interacting with nearby or distant genes (Signal et al., 2016). Furthermore, lncRNAs could regulate the gene expressions in mammalian development.

Intergenic lncRNAs (lincRNAs) are one type of the most widely studied lncRNAs, besides other lncRNA types, such as sense lncRNAs, antisense lncRNAs and intronic lncRNAs (St Laurent et al., 2015). Studies have shown that lincRNA tends to be located on functional elements, such as enhancers and promoters. Enhancers are the short DNA regions which could be bound by activators to enhance the transcription of the target genes in developmental patterns, cell differentiation, even in human diseases (Hnisz et al., 2013; Emera et al., 2016; Karnuta and Scacheri, 2018). Many studies have discovered and described the functional regulations of enhancers on gene expression (Cinghu et al., 2017; Catarino and Stark, 2018). LincRNAs, whose genomic location are overlapped with enhancers, generally transcribe from enhancer regions, and are often named as enhancer-associated lincRNAs (elincRNAs). Thus, they are also considered as enhancer RNAs (eRNAs), and could be involved in the enhancer-promoter looping inside topological associated domains (TAD) (Vance and Ponting, 2014). ElincRNAs could perform enhancer-like function that spatially and temporally regulate the target gene expression in cis or in trans format, during the mammalian development and diseases (Sakabe et al., 2012; Lam et al., 2014; Long et al., 2016). Hon et al. identified the e-lncRNAs from 27919 human lncRNA genes with high-confidence 5′ ends (Hon et al., 2017). More and more studies have shown that elincRNAs play key regulatory roles in cell differentiation and embryonic development. MyoD is a major regulator for muscle differentiation, and Mousavi et al. found that eRNA from the core enhancer could promote the MyoD expression (Mousavi et al., 2013). Recent researches indicated that elincRNAs were indispensable for the gene regulatory network by establishing and stabilizing the chromatin loops of enhancer-promoter interactions (Plank and Dean, 2014; Kim T. K. et al., 2015; Bose et al., 2017). Generally, there is another important type of RNA, named as canonical lincRNAs [also known as promoter-associated lincRNAs (plincRNAs)], presenting the canonical promoter-specific features with H3K4me3 enriched in their TSS intervals (Vance and Ponting, 2014; Kim T. K. et al., 2015).

Using epigenetic features, elincRNAs could be distinguished with the canonical lincRNAs (Signal et al., 2016). ElincRNAs are marked with high H3K4me1 and low H3K4me3 in the TSS regions, which are the enhancer-specific signatures, while canonical lincRNAs are marked with the high H3K4me3 and low or lacked H3K4me1 in the TSS regions which are canonical promoter features (Vance and Ponting, 2014). ElncRNAs and canonical lncRNAs were classified by H3K4me1/H3K4me3 ratio in TSS intervals in human monocytes by Ilott et al. (Ilott et al., 2014). Brain region-specific intergenic or intronic enhancer RNAs were marked with H3K4me1 and H3K27ac enrichment transcribed from enhancers in human genome. Bogu et al. also identified the elncRNAs and plncRNAs (marked by promoter features) across various tissues of mouse, by using the ChromHMM method to interrogate the chromatin status of enhancer and promoter regions (Bogu et al., 2015). Previous studies have shown that elincRNAs could be characterized by high H3K4me1/H3K4me3 ratio in their TSS intervals. As the key regulators for the establishment of the chromatin looping and activation of gene expression, the knowledge about genomic and epigenomic characteristics of elincRNAs is far from completeness. Moreover, H3K36me3 could enrich in the body regions of canonical lincRNAs, while lack in the elincRNAs body regions, just like the enhancers (Natoli and Andrau, 2012; Li et al., 2016). ElincRNA TSS intervals are CGI (CpG Island)-poor regions, while canonical lincRNA TSS intervals are the CGI-rich regions (Li et al., 2016). Thus, elincRNAs could be distinguished from canonical lincRNAs by integrating multi-omic features. However, there are few other known discriminatory chromatin modification or genomic features for the elincRNAs and canonical lincRNAs. DNA methylation could regulate H3K27ac at enhancer regions in mouse ESCs (King et al., 2016), and DNA methylation at enhancers could also identify distinct breast cancer lineages (Fleischer et al., 2017). However, whether this is a specific feature for elincRNAs is still unknown. Furthermore, which features could be contributing factors for elincRNAs have not been systematically interrogated from the perspective of genome, epigenome and regulatome. ElincRNAs could be better characterized by the integration of the significant genomic and epigenomic features. Furthermore, the annotation atlas of elincRNAs could be more comprehensive and complete.

As the acknowledged model organism, mouse is the well-known model for the researches of mammalian development and human diseases. ESCs are the primitive cells derived from preimplantation embryos that have the potential to differentiate into numerous specialized cell types. In this study, chromatin modification data and genomic features of mouse ESCs were collected derived from public online source to identify elincRNAs and canonical lincRNAs based on regularized regression model. Subsequently, elincRNAs and canonical lincRNAs were identified in the genome wide, using the predictive model with the specific features. Further, elincRNAs and canonical lincRNAs were characterized and compared from transcript structure, sequence features, epigenetic modifications and so on. Moreover, it is found that the TF binding patterns of the elincRNAs were different from canonical lincRNAs, which were enriched with specific development associated TFs. The identification of characteristic features of elincRNAs and canonical lincRNAs and the prediction of the two kind lincRNAs might be helpful for the target gene expression regulation of enhancers and their transcriptions in development and human diseases.

## Materials and Methods

### Epigenome, Regulatome, and Transcriptome Datasets

We collected epigenome, regulatome and transcriptome data of mouse ESCs. For epigenome data, the publicly available data contain 12 histone modifications, including 9 active histone modifications and 3 repressive histone modifications (Details see Supplementary Table 1). BS-Seq and DNase-Seq data of mouse ESCs were derived from GEO (Supplementary Table 1). For regulatome data, the 25 TF ChIP-seq data of mouse ESCs were obtained from public repository GEO (Details haven shown in Supplementary Table 1). For transcriptome data, the RNA-Seq data was derived from GEO with the ID GSE39619, and was used to quantify the expression levels of lincRNAs in mouse ESCs. The CAGE data was obtained from FANTOM5 project (http://fantom.gsc.riken.jp/5/).

### Publicly Available Genomic and Functional Annotations

The genome annotation of known lincRNAs were derived from GENCODE Release M6 (GRCm38.p4), and was converted into mm9 assembly version, using NCBI remap_api.pl (https://www.ncbi.nlm.nih.gov/genome/tools/remap/docs/api). CGI and repeat elements annotations of mouse (mm9 version) were obtained from UCSC database. Mouse mm9 reference genome was sourced from UCSC. Conservation scoring by phyloP (phylogenetic *p*-values) for 20 placental mammal genomes, which contained mouse rat, pig, guinea, rabbit, human, chimp, and so on (Details see Supplementary Table 2). Enhancer annotation was derived from vista enhancer database, which contained 568 mouse enhancers (mm9 version) and 1747 human enhancers (hg19 version) (Visel et al., 2007). Promoter annotation was sourced from EPD (The Eukaryotic Promoter Database), in which 21239 mouse promoters (mm9 version) and 23248 human promoters (hg19 version) (Supplementary Table 2) (Dreos et al., 2017).

### Sequencing Reads Alignment

Next Generation Sequencing data in SRA format, including RNA-Seq, ChIP-Seq and BS-Seq, were converted into fastq format by the NCBI-provided program sartoolkit. Trim_galore was used to remove the adapter sequence and low-quality reads of NGS data, and the quality control was performed by FastQC. The data passed the examination of quality control were mapped to the mouse reference genome for the following analysis. RNA-Seq data of mouse ESCs were processed and mapped into mouse reference genome (mm9 version) by HISAT2 (Kim D. et al., 2015), and the expression levels of the annotated and putative lincRNA sets were estimated by StringTie (Pertea et al., 2016). ChIP-Seq data of Histone modifications and TFs were aligned into mouse reference genome (mm9 version) using bowtie2 (Langmead and Salzberg, 2012). Peak callings which identified the enriched signal regions for ChIP-Seq were performed by MACS2 (Zhang Y. et al., 2008). BS-Seq for mouse ESCs was processed by Bismark, and the DNA methylation levels (βvalues) were extracted by bismark_methyaltion_extrasctor at single base resolution (Krueger and Andrews, 2011). The conversion of SAM to BAM file formats was conducted by samtools. The visualization of NGS data was performed by ngs.plot (Loh and Shen, 2016) and deepTools2 (Ramirez et al., 2016), including the meta profiles and the heatmaps of TSS, transcripts and the particular regions. In addition, the K-means clustering based on histone modifications was performed by DeepTools2 (Ramirez et al., 2016).

### Predictive Model for elincRNAs and Canonical lincRNAs

In order to identify elincRNAs, we propose a multi-step predictive method. Firstly, by using H3K4me1 and H3K4me3 signals of TSS intervals, High confidence elincRNAs and canonical lincRNAs were identified as training set for the predictive model. Then, genomic, epigenomic, and regulatory features were calculated for high confidence set. Using the mutil-omic features, the predictive model was developed, based on regularized regression model. Further, the predictive model was evaluated by ten-fold cross-validation and independent test set with high assessment measures. Finally, elincRNAs were identified from the canonical lincRNAs by the predictive model with the specific features.

### Identifying High Confidence Sets of Elincrnas and Canonical Lincrnas

For identifying high confidence sets of elincRNAs and canonical lincRNAs, TSS intervals of elincRNAs and canonical lincRNAs are defined as the regions from TSS up-stream 500 bp to TSS down-stream 500 bp. Gene body regions of elincRNAs and canonical lincRNAs are defined as the regions from TSS down-stream 500 bp to TTS (Transcription Termination Site). Then, 4,157 lincRNA transcripts were collected from the annotation of UCSC mm10 version, whose genomic coordinates were converted to the mm9 version. As significant makers for elincRNAs and canonical lincRNAs, H3K4me1 and H3K4me3 status in the expressed lincRNA TSS intervals were interrogated. Here, the expressed lincRNAs were defined as those with FPKM ≥ 0.5. For a certain expressed lincRNA with at least one histone modification signal ≥10, the H3K4me1/H3K4me3 count ratios in the TSS intervals were calculated. Furthermore, the read count for these two histone modifications were permutated, and random H3K4me1/H3K4me3 ratios were calculated. This process was repeated 10000 times. LincRNAs with the observed H3K4me1/H3K4me3 ratio more than 95 or <5% rank in the random ratio distribution were considered as high confident lincRNAs and canonical lincRNA, respectively.

### Construction of Feature Sets

#### Genomic Features

CGI (CpG Island) and 7 repeat elements of mouse annotations in mm9 version were collected from UCSC (Details are shown in Table 1 and Supplementary Table 1). The coverage ratio of CGI and repeat elements for TSS intervals and gene body regions of lincRNAs in training set were calculated as the genomic features for model training.

**Table 1:** Genomic, epigenomic and regulatomic data used in the prediction model.

**Features**	**Cell type**	**Scource**	**Data type**
Histone modifications (Active modifications: H3k4me1, H3k4me2, H3k4me3, H3k9ac, H3k27ac, H3k36me3, H3k79me2, H3k64ac, H3k122ac, Repressive modifications: H3k9me3, H3k20me3, H3k27me3)	ESCs	GEO	ChIP-Seq
DNA methylation	ESCs	GEO	ChIP-Seq
Repeat elements (DNA, LINE, SINE, LTR, Low complexity, Satellite, Simple repeat)	–	UCSC mm9	Bed format
CpG Island	–	UCSC mm9	Bed format
TFs (Pol2, NelfA, Spt5, Ctr9, Smad1, E2f1, Tcfcp2I1, CTCF, Zfx, STAT3, Klf4, Esrrb, c-Myc, n-Myc, GFP, p300, Suz12, nanog, Oct4, Sox2, Smc1, Smc3, Med12, Med1, Nipbl)	ESCs	GEO	ChIP-Seq

#### Epigenomic Features

DNA methylation and 12 histone modifications for mouse ESCs were collected for predictive model training (Details are shown in Table 1 and Supplementary Table 1). For DNA methylation, the average β value for the probes in TSS intervals and gene body regions were calculated, respectively. The coverage ratio of each histone modification was also be calculated by using the read mapped in TSS intervals and gene body regions of training lincRNAs. DNA methylation and histone modifications were used as the epigenomic features with the value range from 0 to 1.

#### Regulatory Features

We also collected 25 TF ChIP-Seq data of mouse ESCs (Details are shown in Table 1 and Supplementary Table 1). And for the regulatory features, TF binding coverage ratio for TSS intervals and gene body regions were calculated by the reads mapped in the relevant regions, respectively.

### Building the Predictive Model Based on Regularized Regression

The predictive model for elincRNAs was built based on regularization regression model. Regularization regression model could avoid the over-fitting by penalizing high-valued regression coefficients, and which is also known as the shrinkage method. It is important for suppressing over-fitting problems in machine learning. Moreover, there are many characteristic variables in the regression model that do not contribute to the response variables. Therefore, feature selection is required for the predictive model. The regularization regression function was as follows (Equation 1):

(1) $$\begin{array}{r} \sum_{i=1}^{n}y_{i}=\beta_{0}+\sum_{j=1}^{P}\beta_{j}x_{ij} \end{array}$$

Where *y*_*i*_ represents the i th inputted lincRNA. When *y*_*i*_ > 0, the lincRNA was considered as elincRNA; if *y*_*i*_ < 0, the linRNA was considered as canonical lincRNA.*x*_*ij*_ represents the j th feature of the i th inputted lincRNA (*y*_*i*_), and β_*j*_ (*j* = *1,…,p*) represents the contribution degree of the j th feature for the inputted lincRNA. If β_*j*_ > 0, it means that the j th feature is contributing factor for elincRNAs. Otherwise, it means that the j th feature is a contributing factor for canonical lincRNAs.

The regularization algorithm uses a cyclical coordinate descent method to obtain an optimized objective function *L*(β) by continuously optimizing each parameter and iterating until convergence. The objective function *L*(β) (Equation 2) is defined as combination of loss function [Residual Sum of Squares (*RSS*), Equation 3] and penalty term *P*_α_(β) (Equation 4), which both named as penalized residual sum of squares (PRSS).

(2) $$\begin{array}{r} L\left(\beta \right)=RRS+P_{\alpha }\left(\beta \right) \end{array}$$

(3) $$\begin{array}{r} RSS=\sum_{i=1}^{n}{\left(y_{i}-\beta_{0}-\sum_{j=1}^{P}\beta_{j}x_{ij}\right)}^{2} \end{array}$$

(4) $$\begin{array}{r} P_{\alpha }\left(\beta \right)=\sum_{j=1}^{P}\left[\frac{1}{2}\left(1-\alpha \right)\beta_{j}^{2}+\alpha |\beta_{j}|\right] \end{array}$$

The objective function *L*(β) is minimized to estimate the vector of regression coefficients β_*j*_*, j* = *1,…,P*.

A norm, L1–norm (L1 norm regularization), L2–norm (L2 norm regularization) or the combination of L1-norm and L2-norm could be added to the loss function of the regularization method as the penalty term. When α = 1, the penalty term L1-norm is added into loss function, and the regulated regression model is lasso regression with the objective function *L*(β) as follows (Equation 5):

(5) $$\begin{array}{l} L^{lasso}\left(\beta_{1},\ldots ,\beta_{P}\right)=\sum_{i=1}^{n}{\left(y_{i}-\beta_{0}-\sum_{j=1}^{P}\beta_{j}x_{ij}\right)}^{2}\\ \;+\lambda_{1}\sum_{j=1}^{P}{|\beta }_{j}| \end{array}$$

If α = 0, L2-norm penalty is added into loss function, and the regulated regression model is Ridge regression whose objective function *L*(β) as follows (Equation 6):

(6) $$\begin{array}{l} L^{ridge}\left(\beta_{1},\ldots ,\beta_{P}\right)=\sum_{i=1}^{n}{\left(y_{i}-\beta_{0}-\sum_{j=1}^{P}\beta_{j}x_{ij}\right)}^{2}\\ \;+\lambda_{2}\sum_{j=1}^{P}\beta_{j}^{2} \end{array}$$

If 0 < α < 1, combination of L1- and L2- norm is added into RSS, and the regulated regression model is elastic net regression (Equation 7).

(7) $$\begin{array}{l} L^{elastic-net}\left(\beta_{1},\ldots ,\beta_{P}\right)=\\ \sum_{i=1}^{n}{\left(y_{i}-\beta_{0}-\sum_{j=1}^{P}\beta_{j}x_{ij}\right)}^{2}+\lambda_{1}\sum_{j=1}^{P}{|\beta }_{j}|+\lambda_{2}\sum_{j=1}^{P}\beta_{j}^{2} \end{array}$$

Through the addition of a norm penalty, the model could obtain the optimal process of solving the function, thus prevent the over-fitting phenomenon and perform the feature selection. The best combination of contributing factors for elincRNA identification could be prioritized with the lowest mean squared error. In this study, the predictive model for elincRNAs based on regularization regression was performed by R package Glmnet (https://cran.r-project.org/). The selection of α value for regularized regression model was determined by the minimized the error value which generated by cross-validation.

### Model Evaluation

The 10-fold cross-validation and independent testing set were used to estimate the robustness of the predictive model. Training set was divided into 10 equal sized subsets, and 9 subsets were used as training set for model building, while the remaining one subset was used as the validation data for testing the model. This process was repeated for 10 times, in which each single subset was used as the validation data. Moreover, the ROC (Receiver Operating Characteristic) curve and PR (Precision-Recall) curve were drawn, respectively. ROC curve was drawn by plotting the sensitivity (also named as true positive rate, or recall) against the 1-specificity (also known as false positive rate) at various threshold settings. The PR (Precision-Recall) curve was performed by measuring the precision (positive predictive value) against sensitivity at various threshold settings. The AUC (area under the curve) values of ROC curve and PR curve were calculated, which were used to estimate the classification effect of the model. The closer the AUC value is approached to 1, the better performance of the prediction model gets.

### The Independent Testing Set

To collect a comprehensive enhancer list, the vista enhancers of human and mouse were obtained. The genomic sequences of human enhancers were aligned to mouse genome (mm9) using blat program with the threshold 0.85. All the annotations of mouse genomic and the sequence conversed enhancers were overlapped with mouse lincRNAs, expected for the lincRNAs which identified as the high confidence set. And the lincRNAs covering more than half of an enhancer were considered as the elincRNAs. In the same method, the EPD promoter annotations of human and mouse were collected, and the human promoter sequences were aligned into mouse genome (mm9). The collected promoter set was compared with the mouse lincRNA set, and the lincRNAs which were overlapped with more than half of the collected promoters were considered as the canonical lincRNAs. In total, 37 elincRNAs and 69 canonical lincRNAs were obtained and used as the testing set for assessing performance of predictive models.

### Identify Enriched TF Regulations for elincRNAs and Canonical lincRNAs

TF is thought to regulate elincRNAs or canonical lincRNAs, if a certain TF motif is enriched in the corresponding TSS intervals. TF motif data was used to analyze specific transcriptional regulation of elincRNAs and canonical linRNAs. In total, 358 mouse TF binding motif PWMs (Position Weighted Matrix) were collected form HOCOMOCO (HOmo sapiens COmprehensive MOdel COllection) Mouse v11 CORE (Kulakovskiy et al., 2018). AME (Analysis of Motif Enrichment) was used to detect enriched motifs in the TSS regions of lincRNAs with the statistically significance by Fisher's exact test (Bailey et al., 2015). FIMO (Find Individual Motif Occurrences) was used to screen the given TF motifs occurred in the TSS regions of elincRNAs and canonical lincRNAs (Bailey et al., 2015). One elincRNA/canonical linRNA was considered to be regulated by a TF, if this TF motif occurred in the TSS interval of elincRNA/canonical lincRNA, and the ChIP-Seq peak of TF was also observed within elincRNA/canonical lincRNA.

## Results

### Identifying High Confidence Sets of elincRNAs and Canonical lincRNAs

**Figure 1:**
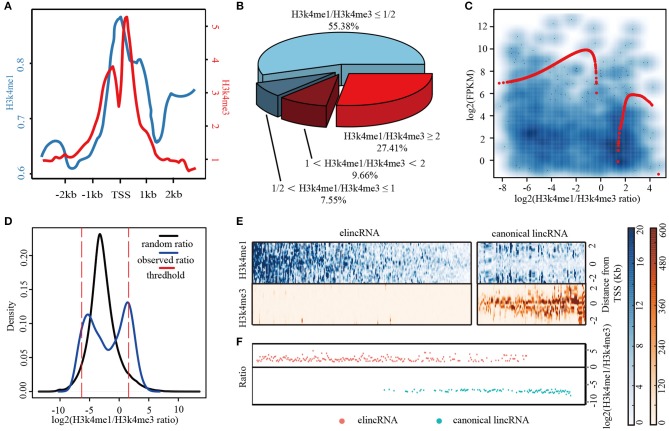
Identification of elincRNAs and canonical lincRNAs with high confidence. **(A)** Density plots showing the distributions of H3K4me1 and H3K4me3 in known lincRNA TSS intervals. **(B)** Analysis of the H3K4me1/H3K4me3 ratio and the corresponding lincRNAs **(C)** Correlation analysis of the H3K4me1/H3K4me3 ratio and expression levels of the corresponding lincRNAs. **(D)** The identification of elincRNAs and canonical lincRNAs. **(E)** Heatmap showing the distributions of H3K4me1 and H3K4me3 in TSS intervals of identified elincRNAs and canonical lincRNAs. **(F)** Dots plots showing the log2 (H3K4me1/H3K4me3) ratio in TSS intervals of identified elincRNAs and canonical lincRNAs.

It is widely acknowledged that, H3K4me1 and H3K4me3 are well-known active chromatin markers for enhancers and promoters, respectively. Thus, the two histone modification markers H3K4me1 and H3K4me3 within TSS intervals of the lincRNAs were interrogated. The average profiles of H3K4me3 and H3K4me1 in TSS intervals of 4,157 annotated lincRNA transcripts in mouse ESCs were shown in Figure 1A, revealing that the lincRNA TSS intervals were enriched by H3K4me3 and H3K4me1 with the pattern of bimodal and unimodal distribution, respectively (Figure 1A). Further, H3K4me1 and H3K4me3 intensities for lincRNA TSS intervals were investigated and shown in Supplementary Figures 1A,B. Unlike those lincRNAs displayed the mRNA-like promoter histone signatures, there was another lincRNA subset marked high H3K4me1 and low H3K4me3, which were enhancer-like histone signatures (Supplementary Figures 1A,B). H3K4me1 and H3K4me3 modification tags were counted in TSS intervals of these lincRNAs. The values of H3K4me1/H3K4me3 ratio were calculated, and the results showed that, more than 50% lincRNA TSS intervals were modified with low H3K4me1/H3K4me3 ratio (Figure 1B), which was consistent with the mRNA-like promoter feature. In addition, 27.41% lincRNAs were modified with high H3K4me1 (H3K4me1/H3K4me3 ≥ 2), which was enhancer signature. More interesting, through analyzing 1284 known lincRNAs with FPKM ≥ 0.5 in mouse ESCs by the LOESS (local polynomial regression) method, we draw the conclusion that the lincRNA expression levels were related with both H3K4me1 and H3K4me3 (Figure 1C). Particularly, canonical lincRNA expression levels might be higher than elincRNAs (Figure 1C). The results above were revealed that, the expressed lincRNAs were associated with the Histone modification H3K4me1 or H3K4me3 enriched in their TSS intervals.

LincRNAs could be divided into enhancer-associated and canonical lincRNAs by chromatin signatures H3K4me1 and H3K4me3 of TSS intervals (Signal et al., 2016). The reads of the two histone modifications in the 1,284 expressed lincRNA TSS intervals were counted for the subsequence analysis (Details were shown in Methods). As the result shown that, a bimodal distribution was presented for H3K4me1/H3K4me3 ratio for the lincRNAs, which was consistent with the results above (Figure 1D). It was revealed that expressed lincRNAs were associated with both H3K4me3 and H3K4me1. For identifying the high confidence sets of elincRNAs and canonical lincRNAs, the signal intensities of H3K4me1 and H3K4me3 for these expressed lincRNAs were permutated, and the random H3K4me1/H3K4me3 ratios were calculated. This process was repeated for 10,000 times, thus, the distribution curve of random H3K4me1/H3K4me3 ratios could be performed with the normal distribution (black line in Figure 1D). The ratio values with the rank of 95 and 5% in the random distribution were used as the thresholds for elincRNAs and canonical lincRNAs, respectively (red dotted lines in Figure 1D) (Details were shown in Methods). By this method, 224 elincRNAs and 112 canonical lincRNAs were identified as the high confidence sets. The chromatin signatures of elincRNAs and canonical lincRNAs were investigated, as expected that, elinRNAs enriched H3K4me1 and depleted of H3K4me3 in TSS intervals (Figure 1E), as well as, H3K4me1/H3K4me3 ratios were >2 (Figure 1F). On the contrary, the canonical lincRNA TSS intervals were marked with low H3K4me1 and high H3K4me3 whose ratio values were <0.5 (Figures 1E,F). The intensity profiles of H3K4me1 and H3K4me3 for elincRNA and canonical lincRNA, was consistent with the previous studies (Supplementary Figures 1C,D).

To estimating the high confident sets of elincRNAs and canonical lincRNAs, two data of chromatin states identified by chromHMM in mouse ESCs were obtained (Yue et al., 2014; Bogu et al., 2015). The high confident elincRNAs or canonical lincRNAs with the coverage more than 0.3 by the relevant chromatin states were considered as the overlapped elincRNAs or canonical lincRNAs, respectively. For the chromatin states of Bogu's research, the number of elincRNAs overlapped by enhancer-like chromatin states were 103 (45.98%), while the of canonical lincRNAs overlapped by promoter-like chromatin states were 109 (97.32%) (Supplementary Figure 2A). Further, the random overlap distributions of elincRNAs and canonical lincRNAs were also acquired, through overlapping the random genomic regions equally with the observed lincRNA transcripts, using the corresponding chromatin state regions in the same criterion. And this process was repeated 10,000 times for Bogu's chromatin state data. It was revealed that the observed overlapped numbers were far from the random overlapped distributions (Supplementary Figure 2A). For Yue's chromatin states data (Yue et al., 2014), elincRNAs and canonical lincRNAs were estimated by the same method. ElincRNAs and canonical lincRNAs overlapped by enhancer- and promoter-like chromatin states were 121 (54.02%) and 112 (100.00%), respectively, which were also far from the random distributions in the same method (Supplementary Figure 2B). Thus, the results indicated that the sets of elincRNAs and canonical lincRNAs identified would be used as the high confidence sets for the subsequence analysis.

### Constructing a Novel Approach to Identify elincRNAs by Integrating Multi-Omic Features

**Figure 2:**
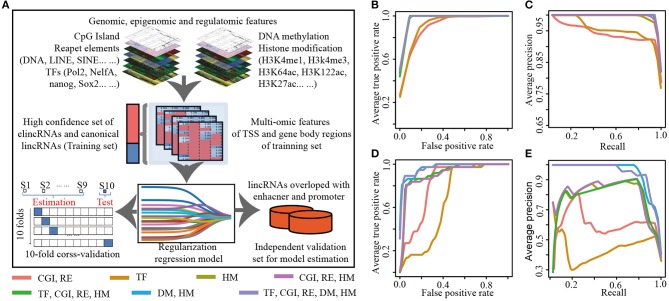
The construction of regularization predictive model **(A)** Workflow of predictive model for identifying elincRNAs. **(B,C)** The average ROC **(B)** and PR **(C)** curves for 10-fold cross validation of the predictive models with different features. **(D,E)** The ROC **(D)** and PR **(E)** curves for the testing set of the predictive models with different features.

For comprehensively characterizing and identifying elincRNAs, we integrated multi-dimensional features to build predictive model for elincRNAs. Genomic, epigenetic and regulatory features were collected, including CGI (CpG Island), 7 types of repeat elements, DNA methylation, 12 histone modifications and 25 TFs derived from the public sources (Details were shown in Table 1 and Supplementary Table 1). Regularization regression model was performed to acquire the best combination of features for predictive model (Figure 2A). The high confident sets of elincRNAs and canonical lincRNAs were used as the positive and negative training set for identifying elincRNAs.

To examine the ability of different omic features to identify elincRNAs, we assessed the performance of predictive models combining distinct features, including sequence features (CGI and repeat elements), epigenomic features and regulatory features. Based on regularized regression model, the predictive models constructed by genomic, epigenomic or regulatory features alone were performed well. The results of 10-fold cross validation for each predictive model were shown in Supplementary Table 3, separately. When 25 TFs were used as the model features, the accuracy was 0.897, which was the lowest value in all of these models. The AUC values of ROC and PR curves were 0.931 and 0.959, respectively (Supplementary Table 3 and Figures 2B,C). The accuracy of the predictive model based on CGI and repeats features was 0.923, which was slightly higher than TFs' model (Supplementary Table 3 and Figures 2B,C). We observed that the model constructed by histone modifications alone and the combination with DNA methylation achieved the accuracies of 0.989 and 0.997, respectively. And the AUC values of ROC and PR curves were all more than 0.99 (Supplementary Table 3). This indicated that the three different features could contribute to identifying elincRNAs. However, it was shown that the TFs might not be the crucial factors for identifying elincRNAs. It was a remarkable fact that, among the TF features, P300 (EP300) which was a co-activator binding to enhancers was not identified as the contributing factor for elincRNAs, possibly because P300 also could be enriched in active promoter regions (Heintzman et al., 2007). Furthermore, genomic characteristics performed better, and the predictive model with epigenomic features performed best (Supplementary Table 3 and Figures 2B,C). Further, to build the predictive model, the different combination of omic features were considered. The model constructed by the combination of genomic features, histone modifications and TFs was performed almost as well as the histone modification alone (accuracy was 0.989) (Supplementary Table 3). Moreover, when removed the TF feature, the predictive model with the combination of genomic and histone modifications performed similarly, with the equal accuracy 0.989. By combining all the features, the performance of the predictive model was a little better. Particularly, the predictive model with histone modification and DNA methylation performed best with the highest accuracy and AUCs values (Supplementary Table 3 and Figures 2B,C). This indicated that epigenomic features including DNA methylation and histone modifications might be crucial for identifying the elincRNAs.

An independent data was also used to estimate the performance of the models (Figure 2A). The vista enhancers of human and mouse were collected to obtain a comprehensive enhancer list (Details were shown in Methods). In total, 37 elincRNAs and 69 canonical lincRNAs were obtained and used as the testing set for assessing the performance of predictive models. For the testing set, the predictive models with the combined features performed better than those with different feature alone (Supplementary Table 4). Predictive models with the combinations of genomic, histone modifications or with addition of the regulatory features got the better performances, with the accuracies of 0.755 and 0.774 respectively (Supplementary Table 4). And their AUCs values of ROC and PR curves were also better than the models with the different omic feature alone (0.668 and 0.444 for model with TF features, 0.815 and 0.608 for model with sequence feature) (Figures 2D,E, and Supplementary Table 4). These results showed the advantages of the integration of multi-omic features. It is noteworthy that the predictive model with the combination of histone modifications and DNA methylation acquired the best performance. And the accuracy achieved 0.859, which was a little higher than the model with all features (the accuracy was 0.858) (Supplementary Table 4). The results revealed that epigenetic features were the crucial signatures for elincRNAs. In brief, by combining DNA methylation and histone modifications, the regularized regression model performed effectively, which represented a novel approach to identify the elincRNAs.

According to the above analysis, the regularized regression model combining the histone modifications and DNA methylation performed best. The appropriate parameter α was interrogated for the optimal predictive model with the combination of epigenetic features. And the results were shown in Supplementary Figure 3, using the model contributing above, when the parameter α of regularized regression model was equal to 1, it was lasso regression model whose parameter λ was equal to 0.0016 with lowest mean squared error 0.0070 (Supplementary Figure 3A). However, the mean squared errors of ElasticNet and Ridge models were all higher than lasso model (Supplementary Figures 3B–D). Thus, when α = 1, lasso model with the DNA methylation and histone modifications was used as the most effective predictive model to identify elincRNAs.

### Both Histone Modifications and DNA Methylation Are Important Features

**Figure 3:**
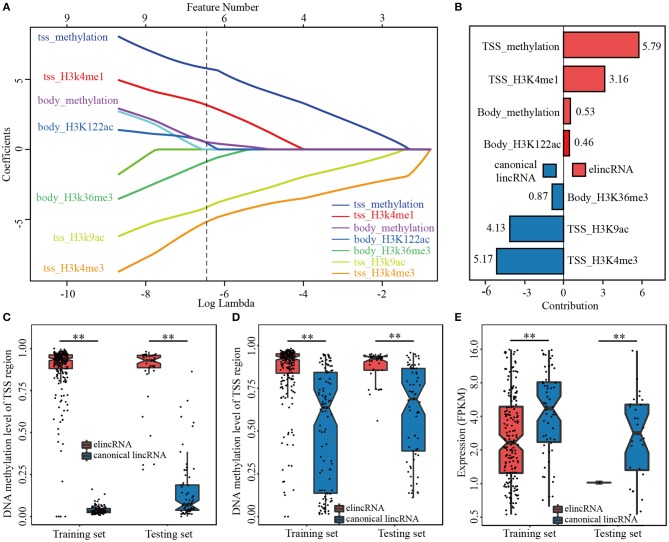
The predictive model for elincRNA identification with the specific features. **(A)** Feature selection of the predictive model for elincRNA identification based on epigenetic features. **(B)** The contribution rates of the identified epigenetic features of predictive model based on the regression coefficients. **(C,D)** DNA methylation levels in the TSS **(C)** and body **(D)** regions of elincRNAs and canonical lincRNAs of training set and testing set in predictive model. **(E)** Expression levels of elincRNAs and canonical lincRNAs of training set and testing set in predictive model. **P < 0.01.

Biased on lasso model with the optimized parameter α, seven specific features among the 26 epigenetic features in TSS and gene body regions of lincRNAs, were identified for elincRNAs and canonical lincRNAs, respectively (Figure 3A). The regression equation, comprised of positive and negative regression coefficients, which represented the different contribution of features for identifying elincRNAs and canonical lincRNAs, respectively (Figures 3A,B) (Details were shown in Methods). The detailed regression equation was as follows:

$$\begin{array}{l} y=2.0860+5.79\times x_{1}+3.16\times x_{2}+0.53\times x_{3}+0.46\times x_{4}\\ \;-5.17\times x_{5}-4.13\times x_{6}-{0.87\times x}_{7} \end{array}$$

In the equation, *x*_1_*, x*_2_, *x*_3_and *x*_4_represented TSS_DNA methylation, TSS_H3K4me1, Body_DNA methylation and Body_H3K122ac, respectively, which were contributing factors for elincRNAs. In addition, *x*_5_*, x*_6_and *x*_7_represented Body_H3K36me3, TSS_H3K9ac and TSS_H3K4me3, which were contributing factors for canonical lincRNAs.

Further, based on the regression coefficients, we interrogated the contribution of the identified features for the predictive model. ElincRNAs were positively relative with TSS_methylation, TSS_H3K4me1, Body_methylation and Body_H3K122ac features (Figures 3A,B). As expected, H3K4me1 in TSS regions was the significant signature to predict elincRNAs whose contribution rate was 3.16. However, the greatest contribution feature for identifying elincRNAs was TSS_methylation with contribution rate 5.79 (Figure 3B). The results showed that DNA methylation of the TSS intervals contributed more to elincRNAs identification than H3K4me1 of the TSS intervals. The body_methylation feature could also be the predictor for elincRNAs, with the contribution rate of 0.53 (Figure 3B). Moreover, the DNA methylation signal intensities of TSS and body regions of elincRNAs and canonical lincRNAs were further compared. Indeed, the average DNA methylation levels of elincRNAs were significantly higher than canonical lincRNAs (Figures 3C,D), which was corresponding with previous results. In the study of Kundaje et al., they showed that the average DNA methylation of active enhancers was significantly higher than that of active TSSs (Roadmap Epigenomics et al., 2015). DNA methylation was enriched in both TSS and body regions of elincRNAs. In TSS and body regions of training and testing sets, the expression levels of elincRNAs were significantly lower than that of canonical lincRNAs, in both training and testing sets (Figure 3E), which was consistent with the expectations. The above results indicated that DNA methylation might be a crucial signature for elincRNAs identification. In addition, histone modification body_H3K122ac, was also identified as the lincRNA-related feature with value of the contribution rate of 0.46 (Figure 3B), which was consistent with recent researches that H3K122ac could mark active enhancers (Pradeepa et al., 2016). However, H3K27ac was not identified as the predictor of elincRNAs in the feature selection process of the predictive model. In the predictive model with histone modifications alone, TSS_H3K27ac was recognized as the significant marker for elincRNAs with the contribution rate of 0.20, which was higher than TSS_H3K122ac (contribution rate of 0.10), but lower than body_H3K122ac (contribution rate of 2.34) and TSS_H3K4me1 (contribution rate of 3.39) (Supplementary Figure 4). This was in accordance with Pradeepa's study that a set of active enhancers was uncovered which was marked by H3K122ac but lack H3K27ac (Pradeepa et al., 2016). Further, the active enhancers marked by H3K27ac were also enriched with H3K122ac (Pradeepa et al., 2016). It was suggested that the predict efficiency of H3K27ac for elincRNAs might be less than TSS-enriched DNA methylation and body-enriched H3K122ac. Thus, the combination of TSS_methylation, TSS_H3K4me1, Body_methylation and Body_H3K122ac could be the significant signatures for elincRNAs.

On the other hand, canonical lincRNAs were relevant with TSS_H3K4me3, TSS_H3K9ac and Body_H3K36me3. As the canonical marker for promoters, TSS_H3K4me3 still had the great significant contribution for the identification of canonical lincRNAs with the contribution rate of 5.17 (Figure 3B). TSS_H3K9ac was also identified as the characteristic feature for canonical lincRNAs with the contribution value 4.13 (Figure 3B), which was an active regulatory marker of with preference for promoters (Consortium, 2012; Roadmap Epigenomics et al., 2015). Moreover, body_H3K36me3, which was an Pol II elongation marker associated with transcribed portions of active genes (Consortium, 2012), was also remarkable for canonical lincRNAs with contribution rate of 0.87 (Figure 3B). In addition to that, H3K36me3 was also found to be associated with active enhancers and be likely to correlate with enhancer RNA transcription (Consortium, 2012), however, H3K36me3 didn't be identified as the effective feature to mark elincRNAs. Therefore, through the regularization regression model, new features were identified to mark elinRNAs (TSS_methylation, Body_methylation and Body_H3K122ac), besides H3K4me1. It could characterize elinRNAs better, and could be helpful for identifying elincRNAs.

### Prediction of elincRNAs Based on Regularization Regression Model

**Figure 4:**
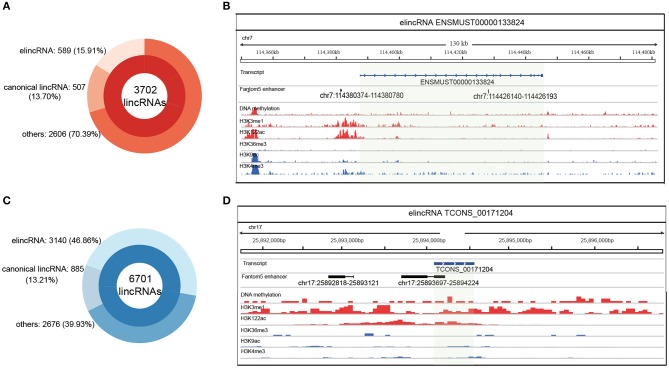
Identification of elincRNAs based on the predictive model with specific epigenetic features. **(A)** Identification of elincRNAs from known lincRNA set by the predictive model. **(B)** Identification of elincRNAs from predicted lincRNA set expressed in mouse ESCs by the predictive model. **(C,D)** Examples for elincRNAs ENSMUST00000133824 **(C)** from known lincRNA set used as training set, and TCONS_00171204 **(D)** from predicted lincRNA set expressed in mouse ESCs identified by predictive model. **(C,D)** Were produced using IGV (Integrative Genomics Viewer), and the green transparent shadows represent the transcript regions of the examples.

For distinguishing more annotations of elincRNAs from canonical lincRNAs, elincRNAs and canonical lincRNAs were identified, using lasso model with the identified epigenetic features. Firstly, among the 3702 known lincRNAs which were not used as training and testing sets for predictive model, 589 elincRNAs and 507 canonical lincRNAs were identified by using the identified epigenetic features (Figure 4A). Moreover, the predictive model was applied to another lincRNA set, which contained 6701 putative lincRNAs in mouse ESCs that we previously identified (Liu et al., 2016). As result, 3140 elincRNAs and 885 canonical lincRNAs were identified (Figure 4B).

An identified elincRNA ENSMUST00000192129 sourced from known lincRNA set, which was located in reverse strand of Chr 1. And the specific epigenetic features were shown in Supplementary Figure 5A. Although H3K4me1 in TSS regions was poor, H3K122ac and DNA methtylation were enriched in the body region with no canonical lincRNA' features. The overlapped transcript ENSMUST00000192129 was an elincRNA identified in training set with the representative features of elincRNAs (Supplementary Figure 5A). Another elincRNA ENSMUST00000133824 used for model training was shown in Figure 4C, which was marked by the elincRNA specific features, and covered with a FANTOM5 annotated enhancer. A putative lincRNA TCONS_00171204 expressed in mouse ESC was identified as an elincRNA with only one exon (Figure 4D). This elinRNA was overlapped with an annotated enhancer with significant epigenetic features (Figure 4D). To mark a contrast with the elincRNAs, a canonical lincRNA ENSMUST00000180932 used for model training was shown in Supplementary Figure 5B, and this lincRNA was marked with high H2K9ac and H3K4me3 in TSS region with de H3K36me3 covered in body region. Thus, we obtained a relative comprehensive elincRNA set for mouse ESCs.

### Characterization of elincRNAs and Canonical lincRNAs

**Figure 5:**
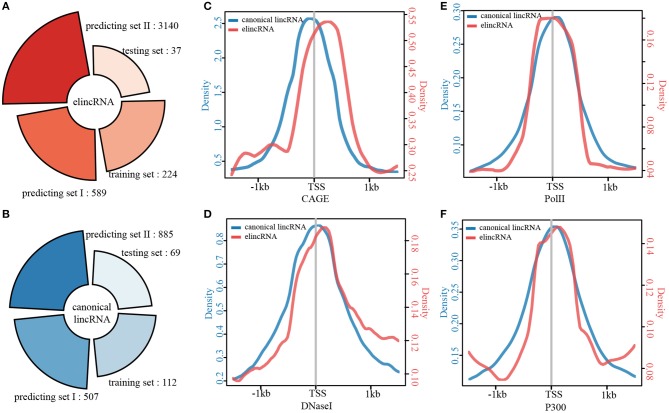
Characterization of elincRNAs and canonical linRNAs. **(A)** ElincRNA set, and **(B)** Canonical lincRNA set, including training set, testing set, and two predicting sets. **(C–F)** The average profiles of CAGE reads **(C)**, DNaseI signals **(D)**, Pol II signals **(E)**, and P300 signals **(F)** around the TSS regions of elincRNA and canonical lincRNA sets.

The results above revealed that elincRNAs could be distinguished from canonical lincRNAs by specific epigenomic features. We detected that if there are any other different features between elincRNAs and canonical lincRNAs. By combining the high confident and predict elincRNAs and canonical lincRNAs, 3990 elincRNAs and 1573 canonical lincRNAs were collected (Figures 5A,B). And then, the elincRNAs and canonical lincRNAs were compared and characterized from various aspects. Firstly, the comparison of transcript length for elincRNAs and canonical lincRNAs were performed and the median values for elincRNAs and canonical lincRNAs were 1490.433 and 1550.179, respectively (Supplementary Figure 6A). It was indicated that the length of elincRNA transcripts was shorter than that of canonical lincRNAs with statistical significance (KS test *p-*value <2.2E-16). Although with no statistical significance, the expression level of elincRNAs was a little lower than that of canonical lincRNAs (Supplementary Figure 6B), whose median values of expression levels were 6.544 and 7.134, respectively.

The genomic features were investigated for elincRNAs and canonical lincRNAs in the following analysis. It has been confirmed that enhancers are generally less conserved (Li et al., 2016), and the conservation of lincRNAs is also significantly lower than protein-encoding transcripts (Derrien et al., 2012). Therefore, it is necessary to interrogate the sequence conservation of exon regions for elincRNAs and canonical lincRNAs. The cumulative probability density distributions of the average conservation for the exon sequences of elincRNA and canonical lincRNA sets were shown in Supplementary Figure 6C. The conservation score of elincRNAs was significantly lower than that of canonical lincRNAs, whose median values were 0.036 and 0.064, respectively. Further, the coverage of CGIs (CpG Islands) in the TSS regions of these two lincRNA sets were compared, and the results were shown in Supplementary Figure 6D. It was found that, canonical lincRNA TSS intervals enriched CGIs with the median coverage 0.237, and elincRNA TSS intervals lacked the coverage of CGIs whose median coverage was only 0.002. Moreover, the CGI coverage of elincRNA TSS regions was much lower than canonical lincRNAs.

The average profiles of CAGE and chromatin modifications in TSS intervals for elincRNAs and canonical lincRNAs were compared. Although the CAGE intensity was significantly lower than that of the canonical lincRNA TSS regions, it still existed a peak in elincRNA TSS intervals (Figure 5C). DNase I hypersensitive sites are acknowledged to be characterized by open accessible chromatin. Thus, the DNaseI average profiles of the TSS regions for the two transcript sets were compared (Figure 5D). Similar results were obtained that elincRNAs and canonical lincRNAs showed a unimodal distribution in the TSS regions, but the enrichment of elincRNAs was significantly lower than that of canonical lincRNAs (Figure 5D). The phenomenon that the low abundances of the active signals in TSS intervals might be related to the universal low expression abundances of elincRNAs. Moreover, we compared PolII and P300 for TSS intervals of elincRNAs and canonical lincRNAs, and the results indicated that elincRNAs and canonical lincRNAs had distinct modification characteristics, respectively (Figures 5E,F).

Moreover, the coverage of repeat elements in TSS intervals were analyzed, including three retrotransposons (LINE, SINE and LTR) (Supplementary Figures 7A–C), DNA transposons (Supplementary Figure 7D), and three tandom repeats (Satellite, Micro-satellite and Mini-Satellite) (Supplementary Figures 7E–G). The coverages of these seven repeat elements in TSS regions were significantly different between elincRNAs and canonical lincRNAs, indicating that the elincRNA TSS intervals might be enriched with repeat elements expect for mini-Satellite (Supplementary Figures 7A–G). Although CGI and repeat elements were not well predictors for identifying elincRNAs, both CGI and repeat elements were significantly different between the two lincRNA sets. We compared the elincRNAs with 680 dbSUPER annotated super-enhancers including 4343 constituents. The result showed that 273 elincRNAs were overlapped with 164 super-enhancers containing 426 constituents (Supplementary Figure 7H). Among the 3990 elincRNAs, 308 were overlapped with CAGE peaks or TSSs predicted by CAGE peaks of FANTOM in both forward and reverse strands, which might be bidirectional transcripts. Summarizing the results above, the elincRNAs and canonical lincRNAs exhibited distinctly specific transcript characteristics, sequence features and chromatin modification features.

### ElincRNAs and Canonical lincRNAs Are Regulated by Distinct TF Regulatory Patterns

Since the distinct sequence features and chromatin modifications around TSS intervals of elincRNAs and canonical lincRNAs, we interrogated the TF regulatory functions on their TSS intervals. Based on known TF motifs obtained from the HOCOMOCO Mouse v11 CORE and JASPAR CORE (2018), the enrichment TF motifs in TSS intervals for elincRNAs and canonical lincRNAs were detected by using AME (Details were shown in Methods).

**Figure 6:**
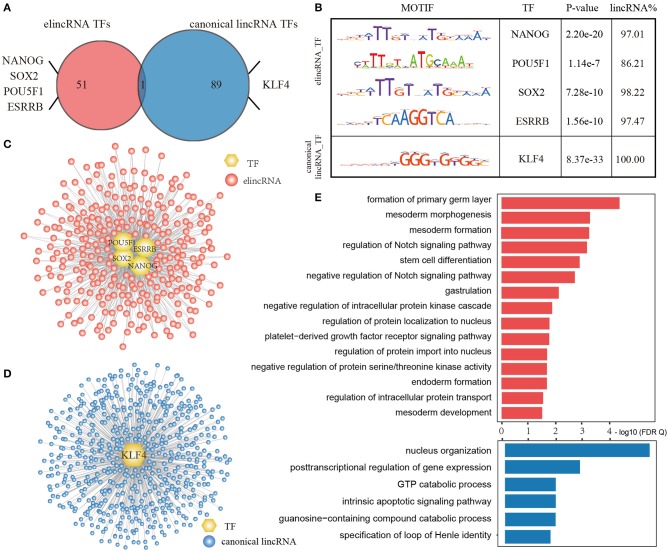
Dissecting the specific transcriptional regulation of elincRNAs and canonical lincRNAs. **(A)** Venn plot for enriched TF motif of elincRNA and canonical lincRNA sets. **(B)** Representative TF motifs in TSS regions of elincRNAs and canonical lincRNAs. **(C)** Specific TF regulatory network for elincRNAs by representative TFs. **(D)** Specific TF regulatory network for canonical lincRNAs by representative TFs. **(E)** The GO BP enrichment for elincRNAs and canonical lincRNAs regulated by representative TFs, respectively.

As a result, 52 TF motifs were enriched in elincRNA TSS intervals, while 90 motifs were enriched in canonical lincRNA TSS intervals with statistical significance (*E-*value < 0.05) (Figure 6A). There were four TFs named as NANOG (Loh et al., 2006), POU5F1 (OCT4) (Zhang X. et al., 2008), SOX2 (Kim et al., 2008) and ESRRB (Festuccia et al., 2018), which were the acknowledged proteins or regulators related to cell differentiation and embryonic development, were enriched in the elincRNA TSS regions. NANOG, POU5F1, and SOX2 were the core markers for stem cells, which were essential to maintain mouse embryonic stem cell pluripotency (Loh et al., 2006; Kim et al., 2008). They bound 97.01, 86.21, 98.22, and 97.47% elincRNAs with the statistical significance for NANOG, POU5F1, SOX2, and ESRRB (Figure 6B). And, KLF4 existed among the canonical lincRNA enriched TF sets with statistical significance, which was a critical regulator for cell reprogramming and early embryonic development in mouse (Ye et al., 2018).

Furthermore, only one TF motif ZN143 was common in both elincRNAs and canonical lincRNAs enriched TF sets (Figure 6A), indicating that elincRNAs and canonical lincRNAs had specific regulatory patterns by binding the distinct TF sets. By integrated the available ChIP-Seq data, we further identified the regulatory relations of the TFs for the elincRNAs and canonical linRNAs, and constructed regulatory network for elincRNAs and caonical lincRNAs, respectively. An elincRNA or a canonical lincRNA was considered to be regulated by a certain TF, if the TSS intervals existed the predicted binding sites and were covered by the corresponding ChIP-Seq peak. The elincRNA regulatory network comprised 662 elincRNAs (Figure 6C), while the canonical lincRNA regulatory network comprised 567 canonical lincRNAs (Figure 6D).

Moreover, function enrichment results found that elincRNAs and canonical lincRNAs were usually involved in the different biological processes, via GREAT method based on the GO BP annotation (Figure 6E, FDR <0.05). For these elincRNAs regulated by ESC markers, they are significantly involved in well-known functions related with stemness maintenance and cell differentiation, including ormation of primary germ layer, gastrulation, the morphogenesis, formation and development of mesoderm, endoderm formation, stem cell differentiation and so on. The elincRNAs were also enriched in several GO BP terms related with Notch signaling pathway, which have been discovered contributing to the formation, growth (Rowan et al., 2008), and development of embryos (Rowan et al., 2008; Fernandez-Valdivia et al., 2011; Djabrayan et al., 2012), even could play crucial functions in the embryonic cell differentiation (Ben-Shushan et al., 2015). While, the canonical lincRNAs regulated by KLF4 tended to regulate basic biological functions, such as nucleus organization, posttranscriptional regulation of gene expression, which were also important for life function maintenance (Figure 6E). Summarizing the results above, elincRNAs and canonical lincRNAs had the specific regulatory patterns, and elincRNAs might be involved in development specific biological processes while canonical lincRNAs played the basic biological functions.

## Discussion

In this study, we developed a novel approach to identify elincRNAs by integrating multi-omic data. We first revealed that expressed lincRNAs could be marked by two common active chromatin modifications H3K4me1 and H3K4me3. And then, several epigenetic features were identified as the signatures for elincRNAs and canonical lincRNAs by lasso regression model. Besides the common acknowledged features H3K4me1 and H3K4me3, more specific features were recognized in our predictive model. For example, DNA methylation and H3K122ac could be the novel signatures to mark elincRNAs, and H3K9ac and H3K36me3 could be the makers for canonical lincRNAs. Unexpectedly, DNA methylation contributed much more than H3K4me1 in the TSS intervals for mouse ESCs. TSS_ and body_DNA methylation were both significant features for elincRNAs, which was consistent with the previous study that the DNA methylation level of active enhancers were commonly higher than active promoters (Roadmap Epigenomics et al., 2015). And in Charlet's study, it was shown that DNA methylation could co-exist with H3K27ac at enhancers and super-enhancers, but not at promoters (Charlet et al., 2016). DNA methylation play important roles in cell differentiation, embryonic development and complex diseases (Su et al., 2018; Yu et al., 2019). Thus, DNA methylation might play unexpected roles at enhancer regions. In our predictive model, H3K27ac was not identified as the significant feature to mark elincRNAs, However, H3K122ac was recognized to enriched in the body regions of expressed elincRNAs, which was consistent with the previous study (Pradeepa et al., 2016). In Pradeepa's study, H3K122ac was identified as a novel signature for active enhancers which were enriched with H3K27ac, and also could mark a subset of active enhancers without H3K27ac enriched (Pradeepa et al., 2016). Thus, H3K122ac might play the important roles in elincRNAs. However, when removing DNA methylation from the predictive model, TSS_H3k27ac could be identified as the contributing factor for elincRNA, despite its contributing score (coefficient) was less than H3K4me1 and H3K122ac. Thus, the combination of features TSS_methylation, TSS_H3K4me1, Body_DNA methylation and Body_H3K122ac enrichment could be the effective markers for elincRNAs. Therefore, the signature sets for elincRNAs and canonical lincRNAs were much more complemented and perfected, than the features H3K4me1 and H3K4m3 alone.

When constructing the predictive model, we used the high confident elincRNA sets identified by the H3K4me1/H3K4me3 ratios. Through the predictive model with identified epigenetic features, we identified 589 expressed elincRNAs from the known lincRNAs, and identified 3140 expressed elincRNAs from the expressed lincRNA set in mouse ESCs (Liu et al., 2016). Along with 224 elincRNAs in training set and 37 elincRNAs in testing set, total 3990 elincRNAs were collected in mouse ESCs. When interrogated the FANTOM5 annotated enhancers, 1179 enhancers were found covered by our identified elincRNAs. We also compared the 3990 elincRNAs with the 147 lncRNAs that were demonstrated to affect the global gene expression in Guttman's study (Guttman et al., 2011), and 48 lncRNAs could be covered by our identified elincRNAs, including 36 known lincRNAs and 58 predicted lincRNAs. Thus, the atlas of elincRNAs depicted by the predictive model provided essential for insight into the regulatory function roles of elincRNAs during the embryonic development.

In the analysis for the specific regulatory patterns of elincRNAs and canonical licnRNAs, we found that, there were specific TF motifs enriched in elincRNAs or canonical lincRNA TSS intervals. Further, the regulatory relationship of several specific enriched TFs were validated by the corresponding ChIP-Seq peaks in mouse ESCs, including NANOG, POU5F1, SOX2 and ESRRB, which were crucial regulators in circuitry controlling ES cell state (Young, 2011). Thus, elincRNAs might be essential component of the TF regulatory circuitry, which were involved in the key regulatory functions of stem cells. Further, the analysis of the elincRNAs and canonical lincRNAs regulated by the specific TFs showed that elincRNAs tended to be involved in the biological processes related with cell differentiation and embryonic development. This indicated that elincRNAs might play the crucial roles in mouse embryonic development.

In conclusion, this work provides a novel approach to identify elincRNAs and canonical lincRNAs by combination of genomic, epigenomic and regulatomic features based on the regularization regression model. Specific epigenetic features were recognized to mark elincRNAs and canonical lincRNAs, respectively. This would help to supplement and improve the atlas of elincRNAs, and dissect the crucial roles of elincRNAs in mouse embryonic development and complex diseases.

## Data Availability Statement

Publicly available datasets were analyzed in this study. This data can be found in the Gene Expression Omnibus, accession numbers: GSE39619, GSE11172, GSE12241, GSE66023, GSE40951, GSE31039, GSE30202, GSE20530, GSE11431, GSE22562.

## Author Contributions

QioW and JX conceived and designed the experiments. HL, TJ, SW, and XC acquired the experiment data. HL, XJ, QiW, and XL performed the study. HL and JY carried out the data analysis. HL wrote this manuscript. JX, YL, and TS revised the manuscript. All authors have read and approved the final manuscript.

### Conflict of Interest

The authors declare that the research was conducted in the absence of any commercial or financial relationships that could be construed as a potential conflict of interest.
