# Supplementary material for: A Novel Approach to Identify Enhancer lincRNAs by Integrating Genome, Epigenome, and Regulatome
Source: Front Bioeng Biotechnol. 2019 Dec 17;7:427. doi: 10.3389/fbioe.2019.00427 (PMC6951418; doi:10.3389/fbioe.2019.00427)
Supplement: Supplementary file 1 [file Table_1.DOCX]

Supplementary Material

# Supplementary Figures and Tables

## Supplementary Figures


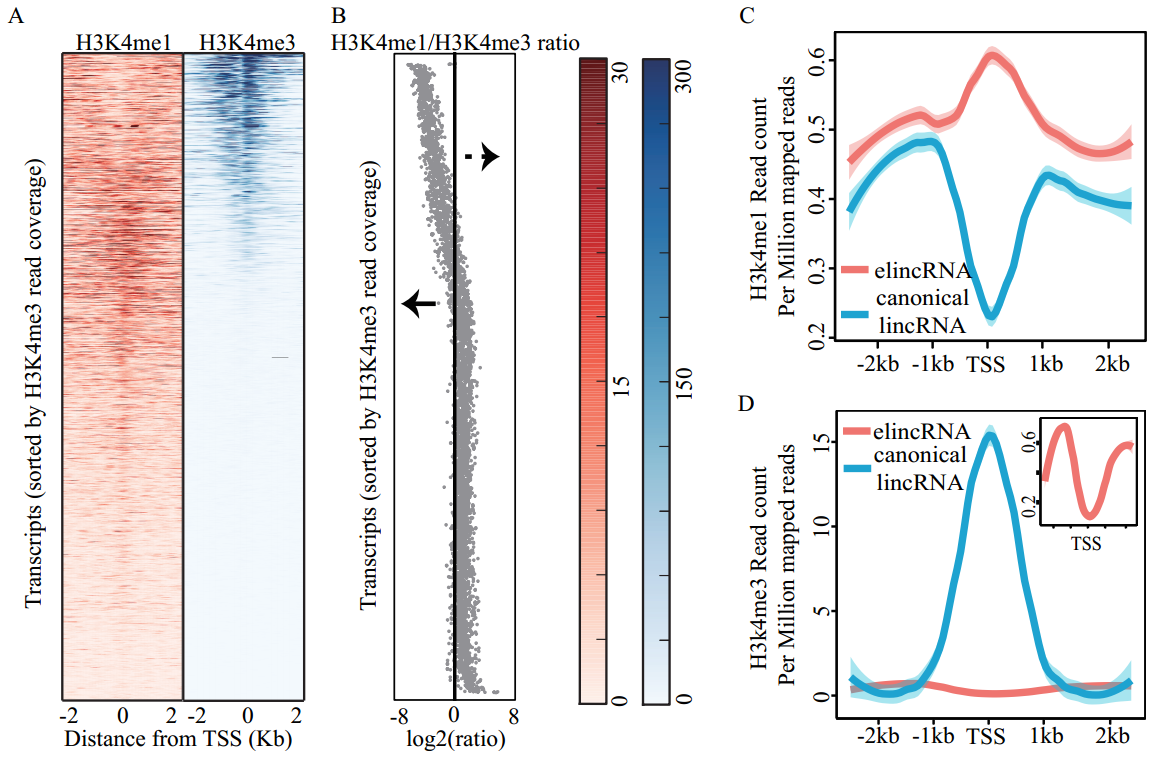


**Supplementary Figure 1.** Representative hitone modifications for elincRNAs and canonical lincRNAs. (A) H3K4me1 and H3K4me3 binding around the TSS regions of lincRNAs. Heatmaps were sorted by the H3K4me3 read coverage. (B) Dot plot of the H3K4me1/H3K4me3 log2(ratio) at each TSS regions. (C-D) The average profiles of H3K4me1 (C) and H3K4me3 (D) reads around the TSS regions of high confident elincRNAs and canonical lincRNAs. Shadow regions in(C) and (D) represent the 5–95% bootstrap confidence intervals of the statistics.


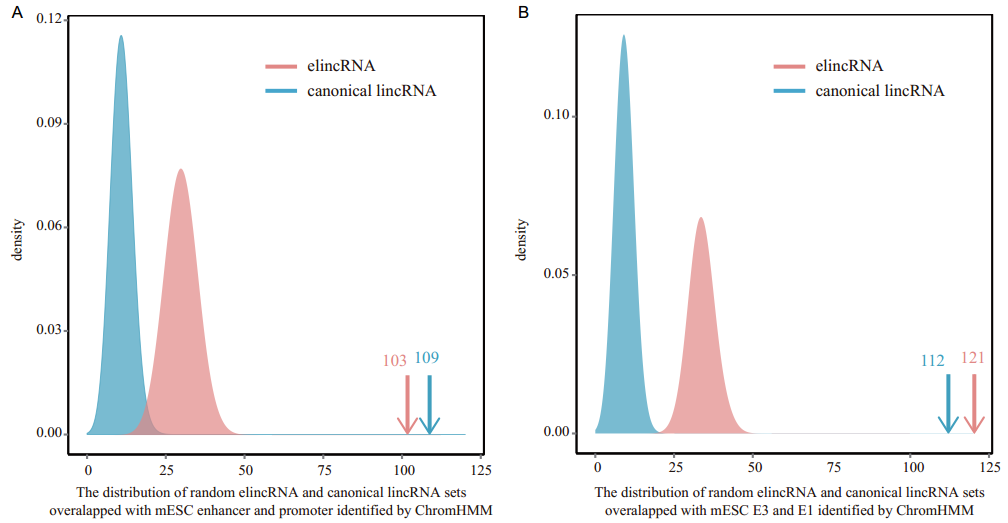


**Supplementary Figure 2.** Estimation of the high confident elincRNAs and canonical linRNAs. (A) The comparison between the random distributions and the observed numbers of elincRNAs and canonical lincRNAs overlapped by enhancer and promoter regions identified by chromHMM. (B) The comparison between the random distributions and the observed numbers of elincRNAs and canonical lincRNAs overlapped by H3K4me1- and H3K4me3- enriched regions identified by chromHMM.


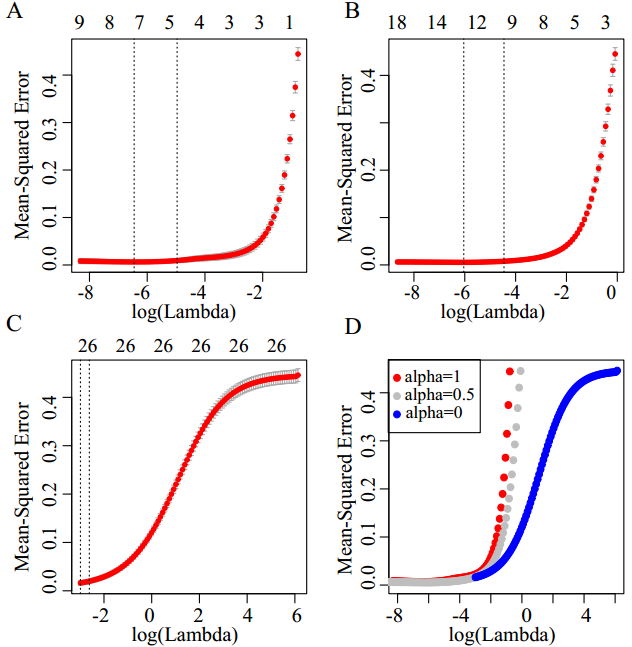


**Supplementary Figure 3.** The parameter selection for optimized predict model. (A)-(C) distributions of the mean squared errors of parameter selection for prediction models with α=1(A), 0.5(B) or 0(C). (D) Comparison of mean squared errors of parameter selection for optimized predict models.


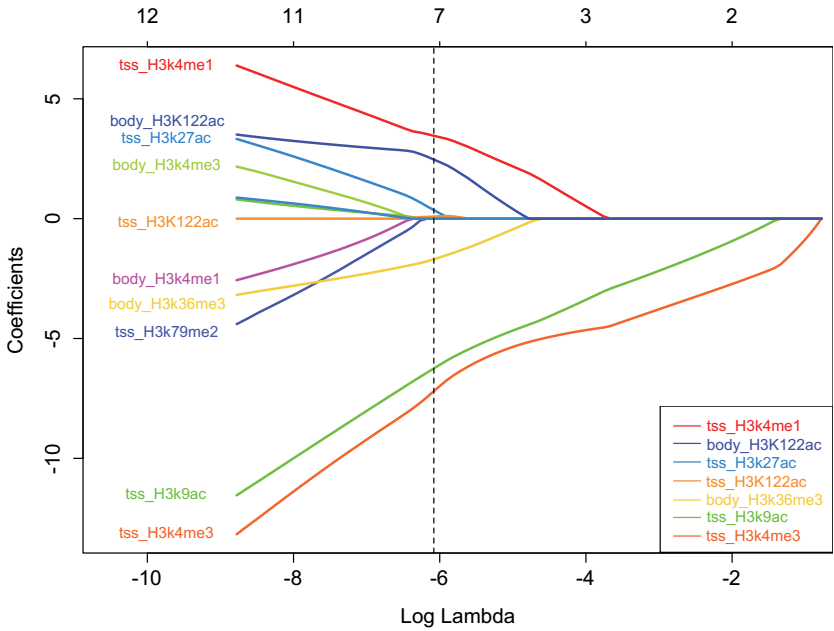


**Supplementary Figure 4.** The predict model for elincRNA identification with histone modifications alone.


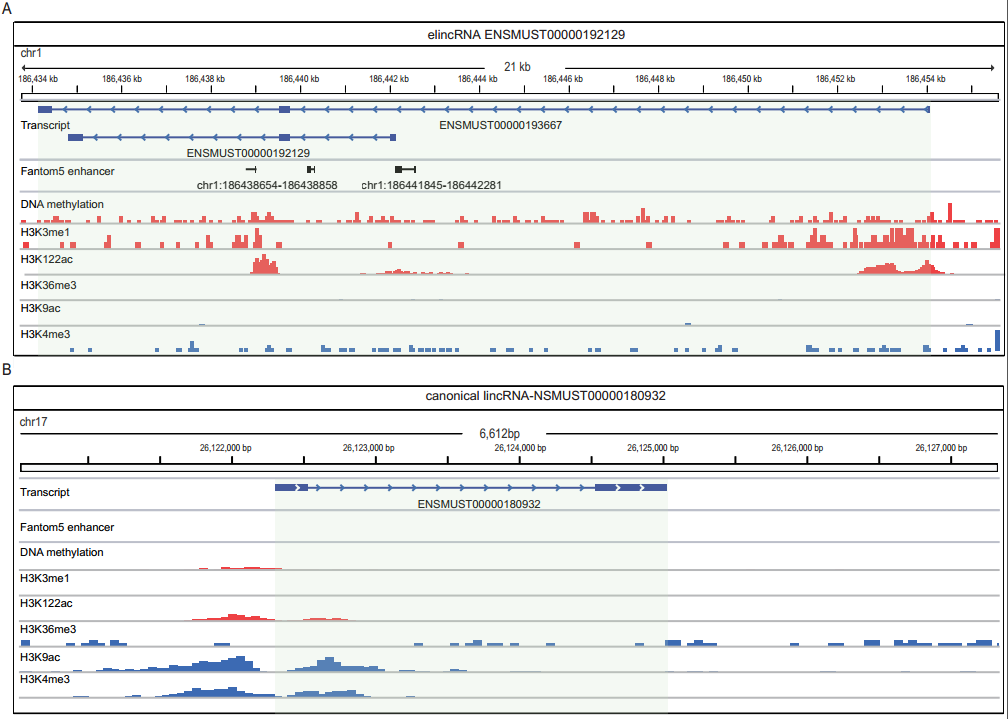


**Supplementary Figure 5.** Examples of elincRNAs and canonical lincRNAs with the Representative chromatin features. (A) Examples of a high confident elincRNA ENSUT000001933667 and a predicted elincRNA ENSMUST00000192129 with the specific histone modification signatures identified with the optimized predict model. (B) An exalple of canonical lincRNA with the promoter-lilke chromatin signatures identified with the optimized predict model. Figures were produced using IGV (Integrative Genomics Viewer), and the green transparent shadows represent the transcript regions of the examples.


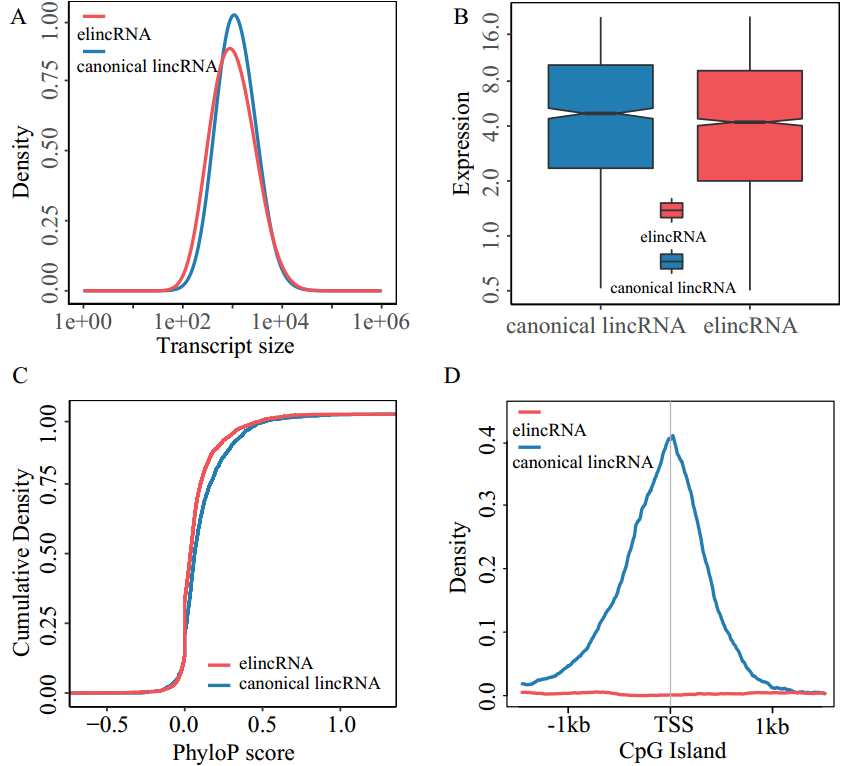


**Supplementary Figure 6.** Genomic characterization of elincRNAs and canonical lincRNAs. The comparision of transcript length (A), expression level (B), conversion score of transcripts (C) and CGI coverage around TSS regions(D) of elincRNAs and canonical lincRNAs.


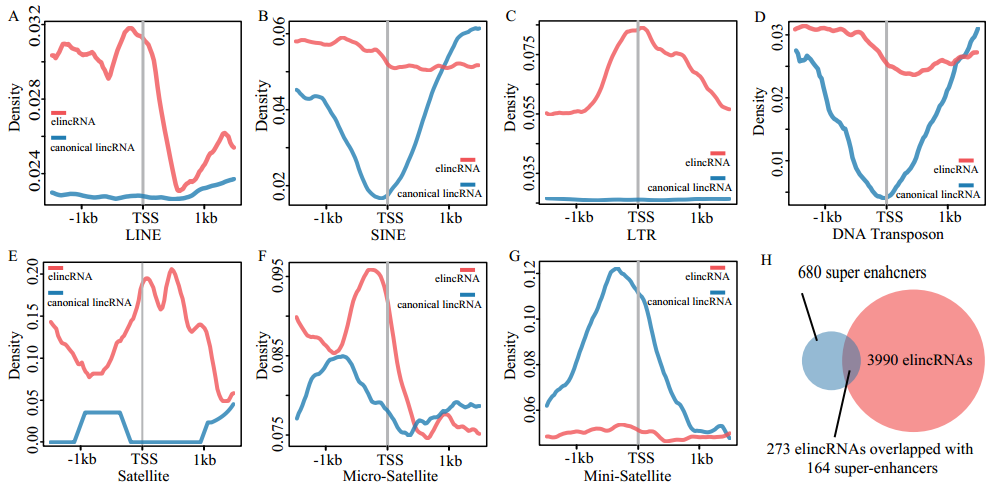


**Supplementary Figure 7.** Characterization of repeat elements around TSS regions of elincRNAs and canonical lincRNAs, including LINE(A), SINE(B), LTR(C), DNA transposon(D), Satellite(E), Micro-Satellite(F) and Mini-Satellite(G). (H) Venn plot showing the relationship between elincRNAs and super enhancers in mouse ESCs.

## Supplementary Tables

**Supplementary Table 1.** The relevant information of the NGS data used in this study

| SRA ID | cell type | GEO ID | GNS type | antibody | layout | length | source |
| --- | --- | --- | --- | --- | --- | --- | --- |
| **Transcriptome data** |  |  |  |  |  |  |  |
| SRX172458 | mESCs | GSE39619 | RNA-Seq | - | PAIRED | 101 | ENCODE |
| **Epigenome data** |  |  |  |  |  |  |  |
| SRX000583 | mESCs | GSE11172 | ChIP-Seq | H3k4me1 | SINGLE | 36 | [[1](#_ENREF_1)] |
| SRX000582 | mESCs | GSE11172 | ChIP-Seq | H3k4me2 | SINGLE | 36 | [[1](#_ENREF_1)] |
| SRX001923 | mESCs | GSE12241 | ChIP-Seq | H3k4me3 | SINGLE | 36/27 | [[2](#_ENREF_2)] |
| SRX001921 | mESCs | GSE12241 | ChIP-Seq | H3k27me3 | SINGLE | 32/27 | [[2](#_ENREF_2)] |
| SRX001922 | mESCs | GSE12241 | ChIP-Seq | H3K36me3 | SINGLE | 32/27 | [[2](#_ENREF_2)] |
| SRX001924 | mESCs | GSE12241 | ChIP-Seq | H3K9me3 | SINGLE | 32/27 | [[2](#_ENREF_2)] |
| SRX001925 | mESCs | GSE12241 | ChIP-Seq | H4K20me3 | SINGLE | 32/27 | [[2](#_ENREF_2)] |
| SRX003860 | mESCs | GSE11724 | ChIP-Seq | H3K79me2 | SINGLE | 26 | [[3](#_ENREF_3)] |
| SRX003859 | mESCs | GSE11724 | ChIP-Seq | H3K79me2 | SINGLE | 26 | [[3](#_ENREF_3)] |
| SRX1560889 | mESCs | GSE66023 | ChIP-Seq | H3k64ac | SINGLE | 42 | [[4](#_ENREF_4)] |
| SRX1560890 | mESCs | GSE66023 | ChIP-Seq | H3k64ac | SINGLE | 50 | [[4](#_ENREF_4)] |
| SRX1560887 | mESCs | GSE66023 | ChIP-Seq | H3k122ac | SINGLE | 42 | [[4](#_ENREF_4)] |
| SRX1560888 | mESCs | GSE66023 | ChIP-Seq | H3k122ac | SINGLE | 50 | [[4](#_ENREF_4)] |
| SRX187620 | mESCs | GSE40951 | ChIP-Seq | H3k9ac | SINGLE | 48 | [[5](#_ENREF_5)] |
| SRX187619 | mESCs | GSE40951 | ChIP-Seq | H3k27ac | SINGLE | 48 | [[5](#_ENREF_5)] |
| SRX085432 | mESCs | GSE31039 | ChIP-Seq | H3k4me1 | SINGLE | 36 | ENCODE |
| SRX085431 | mESCs | GSE31039 | ChIP-Seq | H3k4me3 | SINGLE | 36 | ENCODE |
| SRX080191 | mESCs | GSE30202 | BS-Seq | - | SINGLE | 100 | [[6](#_ENREF_6)] |
| SRX191012 | mESCs | GSE37074 | Dnase-Seq | - | SINGLE | 36 | ENCODE |
| **Regulatome data** |  |  |  |  |  |  |  |
| SRX143841 | mESCs | GSE36027 | ChIP-Seq | Pol2 | SINGLE | 36 | [[7](#_ENREF_7)] |
| SRX017058 | mESCs | GSE20530 | ChIP-Seq | NelfA | SINGLE | 36 | [[8](#_ENREF_8)] |
| SRX017059 | mESCs | GSE20530 | ChIP-Seq | Spt5 | SINGLE | 26 | [[8](#_ENREF_8)] |
| SRX017060 | mESCs | GSE20530 | ChIP-Seq | Ctr9 | SINGLE | 26 | [[8](#_ENREF_8)] |
| SRX000548 | mESCs | GSE11431 | ChIP-Seq | Smad1 | SINGLE | 36 | [[9](#_ENREF_9)] |
| SRX000541 | mESCs | GSE11431 | ChIP-Seq | E2f1 | SINGLE | 26 | [[9](#_ENREF_9)] |
| SRX000551 | mESCs | GSE11431 | ChIP-Seq | Tcfcp2I1 | SINGLE | 26 | [[9](#_ENREF_9)] |
| SRX000540 | mESCs | GSE11431 | ChIP-Seq | CTCF | SINGLE | 36 | [[9](#_ENREF_9)] |
| SRX000552 | mESCs | GSE11431 | ChIP-Seq | Zfx | SINGLE | 26 | [[9](#_ENREF_9)] |
| SRX000547 | mESCs | GSE11431 | ChIP-Seq | STAT3 | SINGLE | 26 | [[9](#_ENREF_9)] |
| SRX000544 | mESCs | GSE11431 | ChIP-Seq | Klf4 | SINGLE | 26 | [[9](#_ENREF_9)] |
| SRX000542 | mESCs | GSE11431 | ChIP-Seq | Esrrb | SINGLE | 26 | [[9](#_ENREF_9)] |
| SRX000553 | mESCs | GSE11431 | ChIP-Seq | c-Myc | SINGLE | 26 | [[9](#_ENREF_9)] |
| SRX000554 | mESCs | GSE11431 | ChIP-Seq | n-Myc | SINGLE | 26 | [[9](#_ENREF_9)] |
| SRX000543 | mESCs | GSE11431 | ChIP-Seq | GFP | SINGLE | 26 | [[9](#_ENREF_9)] |
| SRX000555 | mESCs | GSE11431 | ChIP-Seq | p300 | SINGLE | 26 | [[9](#_ENREF_9)] |
| SRX000550 | mESCs | GSE11431 | ChIP-Seq | Suz12 | SINGLE | 26 | [[9](#_ENREF_9)] |
| SRX000545 | mESCs | GSE11431 | ChIP-Seq | nanog | SINGLE | 36 | [[9](#_ENREF_9)] |
| SRX000546 | mESCs | GSE11431 | ChIP-Seq | POU5F1 | SINGLE | 26 | [[9](#_ENREF_9)] |
| SRX000549 | mESCs | GSE11431 | ChIP-Seq | Sox2 | SINGLE | 26 | [[9](#_ENREF_9)] |
| SRX022688 | mESCs | GSE22562 | ChIP-Seq | Smc1 | SINGLE | 36 | [[10](#_ENREF_10)] |
| SRX022689 | mESCs | GSE22562 | ChIP-Seq | Smc1 | SINGLE | 36 | [[10](#_ENREF_10)] |
| SRX022690 | mESCs | GSE22562 | ChIP-Seq | Smc3 | SINGLE | 36 | [[10](#_ENREF_10)] |
| SRX022691 | mESCs | GSE22562 | ChIP-Seq | Smc3 | SINGLE | 36 | [[10](#_ENREF_10)] |
| SRX022692 | mESCs | GSE22562 | ChIP-Seq | Med12 | SINGLE | 36 | [[10](#_ENREF_10)] |
| SRX022693 | mESCs | GSE22562 | ChIP-Seq | Med12 | SINGLE | 36 | [[10](#_ENREF_10)] |
| SRX022694 | mESCs | GSE22562 | ChIP-Seq | Med1 | SINGLE | 36 | [[10](#_ENREF_10)] |
| SRX022695 | mESCs | GSE22562 | ChIP-Seq | Med1 | SINGLE | 36 | [[10](#_ENREF_10)] |
| SRX022696 | mESCs | GSE22562 | ChIP-Seq | Nipbl | SINGLE | 36 | [[10](#_ENREF_10)] |
| SRX022697 | mESCs | GSE22562 | ChIP-Seq | Nipbl | SINGLE | 36 | [[10](#_ENREF_10)] |

**Supplementary Table 2.** The relevant information of publicly available annotation data used in this study.

| Annotation type | source | URL |
| --- | --- | --- |
| Known lincRNAs | GENCODCE | https://www.gencodegenes.org/mouse/release_M6.html |
| CGI | UCSC | http://hgdownload.cse.ucsc.edu/goldenPath/mm9/database/ |
| Repeat elements | UCSC | http://hgdownload.cse.ucsc.edu/goldenPath/mm9/database/ |
| Mouse Reference genome | UCSC | http://hgdownload.cse.ucsc.edu/goldenPath/mm9/chromosomes/ |
| Conservation score | UCSC | http://hgdownload.cse.ucsc.edu/goldenPath/mm9/phyloP30way/ |
| Enhancers | Vista enhancer database | https://enhancer.lbl.gov/ |
| Promoters | EPD | http://epd.vital-it.ch/ |

**Supplementary Table 3.** Performance evaluation for 10-fold cross validation of the feature selection model for elincRNAs and canonical lincRNAs by different features

| Feature | accuracy | error | sensitivity | specificity | precision | roc.AUC | pr.AUC |
| --- | --- | --- | --- | --- | --- | --- | --- |
| Histone | 0.989 | 0.011 | 0.996 | 0.973 | 0.988 | 0.992 | 0.993 |
| TF | 0.897 | 0.103 | 0.782 | 0.950 | 0.886 | 0.931 | 0.959 |
| GCI, RE | 0.923 | 0.077 | 0.800 | 0.979 | 0.941 | 0.918 | 0.946 |
| CGI, RE, Histone | 0.989 | 0.011 | 0.996 | 0.973 | 0.988 | 0.991 | 0.993 |
| TF, CGI, RE, Histone | 0.989 | 0.011 | 0.983 | 0.974 | 0.988 | 0.998 | 0.999 |
| Histone, DNA methylation | 0.997 | 0.003 | 0.996 | 0.983 | 0.989 | 0.995 | 0.993 |
| All feature | 0.996 | 0.004 | 0.995 | 0.981 | 0.987 | 0.993 | 0.998 |

**Supplementary Table 4.** Performance evaluation for dependent testing set of the feature selection model for elincRNAs and canonical lincRNAs by different features

| Feature | accuracy | error | sensitivity | specificity | precision | roc.AUC | pr.AUC |
| --- | --- | --- | --- | --- | --- | --- | --- |
| Histone | 0.764 | 0.236 | 0.892 | 0.611 | 0.696 | 0.905 | 0.786 |
| TF | 0.632 | 0.368 | 1.000 | 0.487 | 0.435 | 0.668 | 0.444 |
| GCI, RE | 0.717 | 0.283 | 0.973 | 0.554 | 0.580 | 0.815 | 0.608 |
| CGI, RE, Histone | 0.755 | 0.245 | 0.865 | 0.604 | 0.696 | 0.907 | 0.792 |
| TF, CGI, RE, Histone | 0.774 | 0.226 | 0.892 | 0.618 | 0.696 | 0.918 | 0.781 |
| Histone, DNA methylation | 0.859 | 0.141 | 0.946 | 0.729 | 0.812 | 0.971 | 0.944 |
| All feature | 0.858 | 0.142 | 0.945 | 0.727 | 0.811 | 0.970 | 0.941 |

**REFERENCES**

1. Meissner A, Mikkelsen TS, Gu H et al. Genome-scale DNA methylation maps of pluripotent and differentiated cells, Nature 2008;454:766-770.

2. Goren A, Ozsolak F, Shoresh N et al. Chromatin profiling by directly sequencing small quantities of immunoprecipitated DNA, Nat Methods 2010;7:47-49.

3. Marson A, Levine SS, Cole MF et al. Connecting microRNA genes to the core transcriptional regulatory circuitry of embryonic stem cells, Cell 2008;134:521-533.

4. Pradeepa MM, Grimes GR, Kumar Y et al. Histone H3 globular domain acetylation identifies a new class of enhancers, Nat Genet 2016;48:681-686.

5. Teif VB, Beshnova DA, Vainshtein Y et al. Nucleosome repositioning links DNA (de)methylation and differential CTCF binding during stem cell development, Genome Res 2014;24:1285-1295.

6. Stadler MB, Murr R, Burger L et al. DNA-binding factors shape the mouse methylome at distal regulatory regions, Nature 2011;480:490-495.

7. Shen Y, Yue F, McCleary DF et al. A map of the cis-regulatory sequences in the mouse genome, Nature 2012;488:116-120.

8. Rahl PB, Lin CY, Seila AC et al. c-Myc regulates transcriptional pause release, Cell 2010;141:432-445.

9. Chen X, Xu H, Yuan P et al. Integration of external signaling pathways with the core transcriptional network in embryonic stem cells, Cell 2008;133:1106-1117.

10. Kagey MH, Newman JJ, Bilodeau S et al. Mediator and cohesin connect gene expression and chromatin architecture, Nature 2010;467:430-435.
